# Supplementary material for: Structural Characterization of Covalently Stabilized Human Cystatin C Oligomers
Source: Int J Mol Sci. 2020 Aug 15;21(16):5860. doi: 10.3390/ijms21165860 (PMC7461555; doi:10.3390/ijms21165860)
Supplement: Supplementary file 1 [file ijms-21-05860-s001.pdf]

Supporting Information:

Structural characterization of covalently stabilized human cystatin C oligomers

Magdalena Chrabąszczewska[1,2], Adam K. Sieradzan[3], Sylwia Rodziewicz-Motowidło[3], Anders Grubb[4], Christopher M. Dobson[5,7], Janet R. Kumita[5,6]* and Maciej Kozak[1]*

[1]Department of Macromolecular Physics, Faculty of Physics, Adam Mickiewicz University in Poznań, Umultowska 85, 61-614 Poznań, Poland;

[2]current address: Department of Biophysics, Faculty of Physics, University of Warsaw, Pasteura 5, 02-093 Warsaw, Poland; Magdalena.Chrabaszczewska@fuw.edu.pl

[3]Faculty of Chemistry, University of Gdańsk, Wita Stwosza 63, 80-308 Gdańsk, Poland

[4]Department of Clinical Chemistry, Lund University Hospital, S-22185 Lund, Sweden.

[5]Centre for Misfolding Diseases, Department of Chemistry, University of Cambridge, Lensfield Road, Cambridge, CB2 1EW,UK.

[6] current address: Department of Pharmacology, University of Cambridge, Tennis Court Road, Cambridge, CB2 1PD,UK.

*jrk38@cam.ac.uk

*mkozak@amu.edu.pl

[7]C. M. Dobson passed away on 8th September, 2019

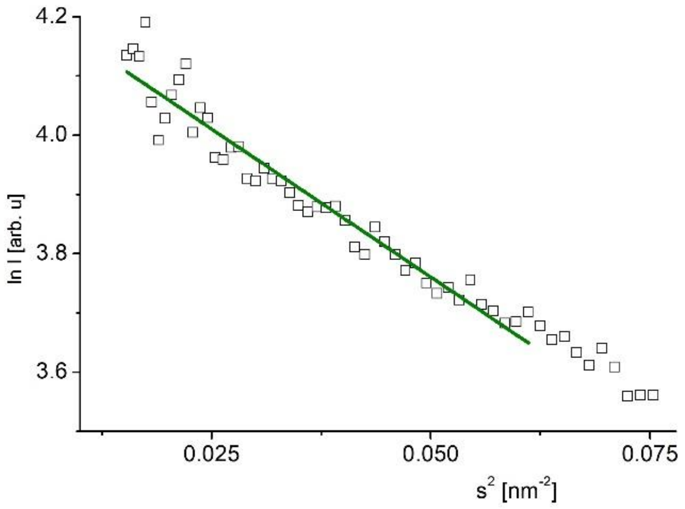

Figure S1. SAXS data recorded for the stab-1 HCC oligomers in solution. Guinier plot of SAXS data collected to define the size of the stab-1 HCC oligomers**.** The fit (green line) to the Guinier equation was performed within the SAXS data range of 0.124 to 0.247 nm^{-1}, where $sR_g < 1.3$ as the particles are assumed to be spherical, resulting in a value of R_g of 5.28 ± 0.3 nm.

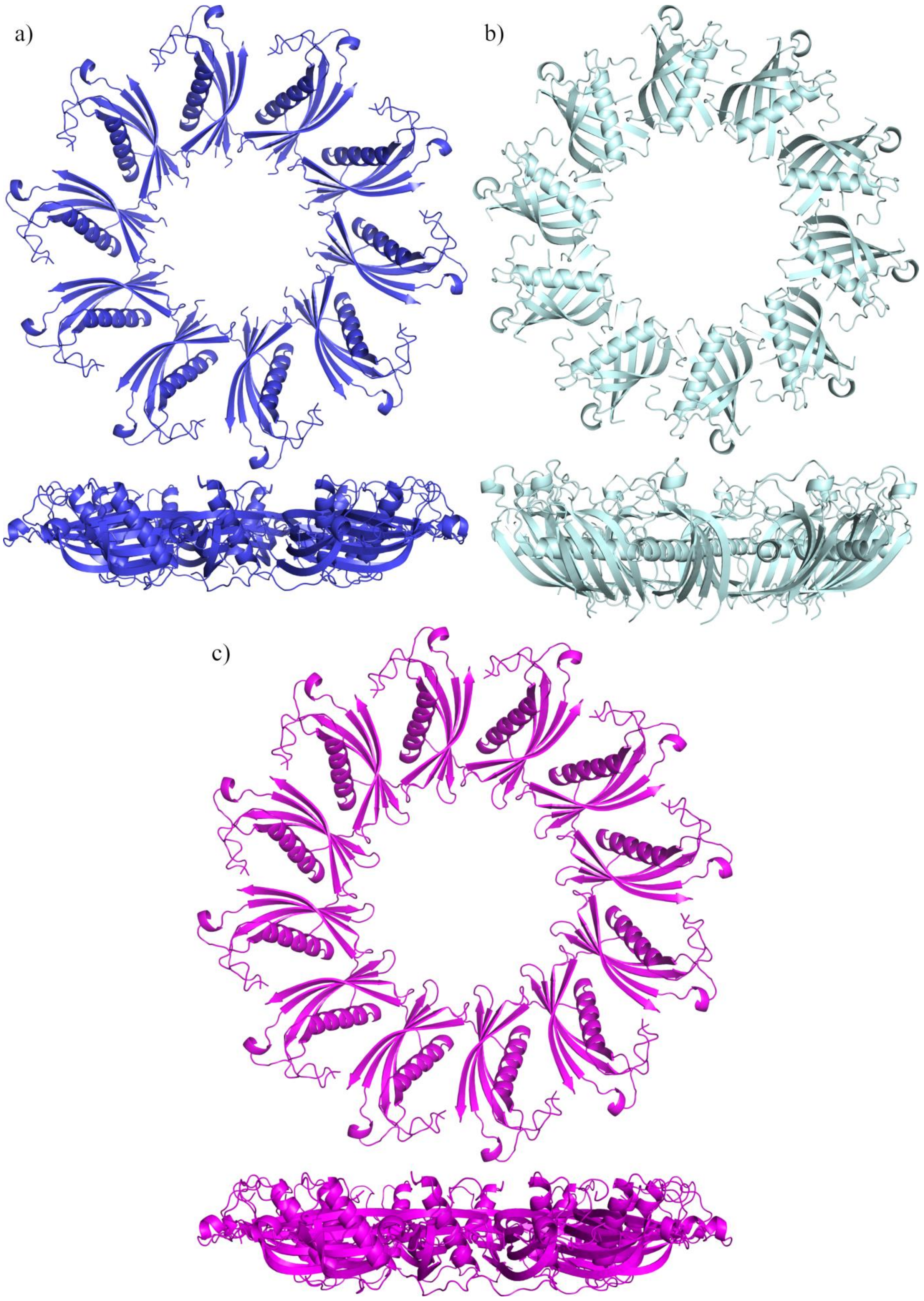

Figure S2. Modelling of the stab-1 HCC oligomers.

Preliminary models selected for MD simulations from set of solutions obtained from SymmDock: **a)** Model 1 of decamer; **b)** Model 2 of decamer; **c)** Model of dodecamer.

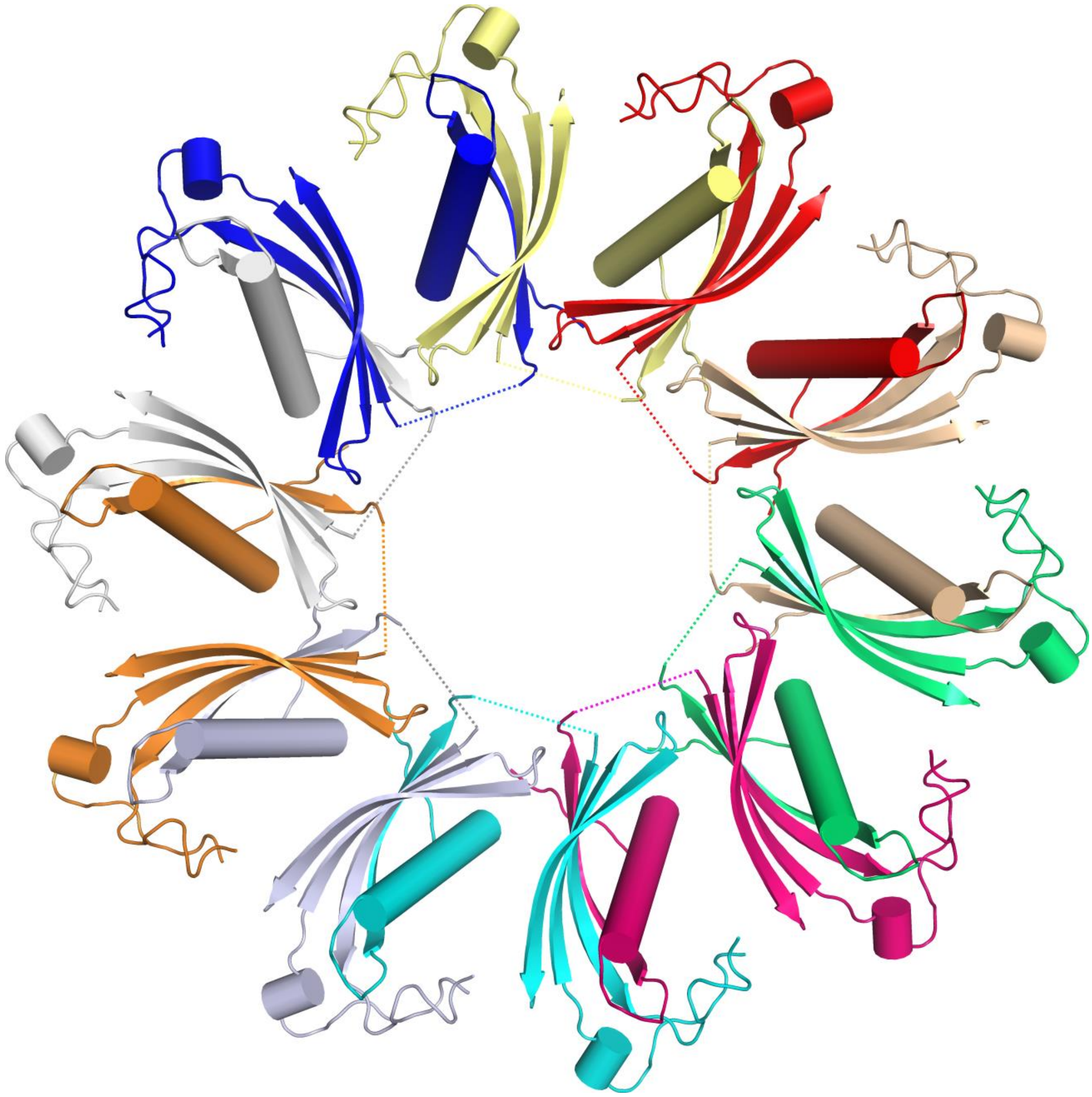

Figure S3. The domain swapping scheme in the stab-1 HCC oligomers.
The domain exchange between adjacent subunits within the stab-1 HCC oligomer from the Model 1 decamer. The colours indicate the individual stab-1 HCC subunits. To implement the domain swap, the bond between residues 58 and59 were manually broken and the atomic positions of the N-terminal fragment of the polypeptide chain (residues up to 58) was transferred from the first stab-1 HCC subunit to the next one, with the rest of the chain remaining unaltered. The dashed lines indicated the broken bond between residues 58 and 59.

a)

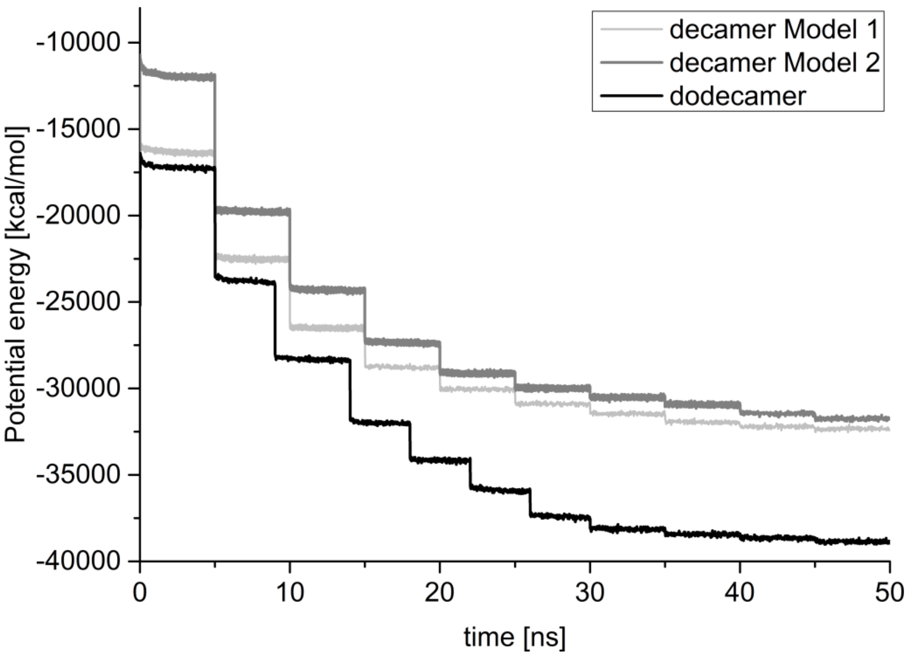

b)

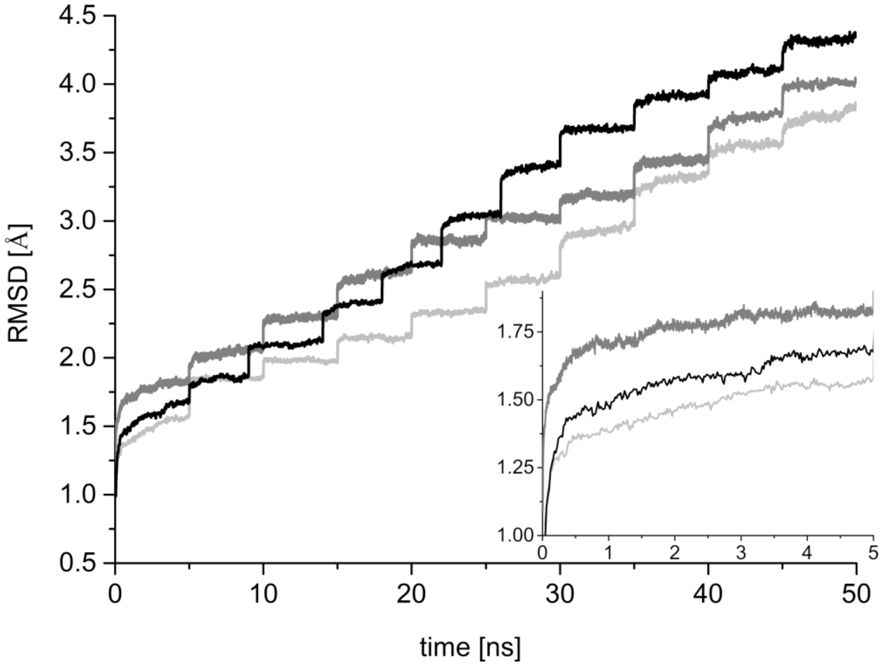

Figure S4. Molecular dynamics simulations of the stab-1 HCC oligomers.
(a) The potential energy (E_{pot}) of the MD system as a function of simulation time for HCC oligomers models. **(b)** The root-mean-square deviation (RMSD) of all C_α atoms calculated by reference to the initial structure taken as a function of the simulation time. (Inset) An expanded view of first 5 ns cycle (light gray - Model 1 of decamer; gray - Model 2 of decamer, dark gray – dodecamer).

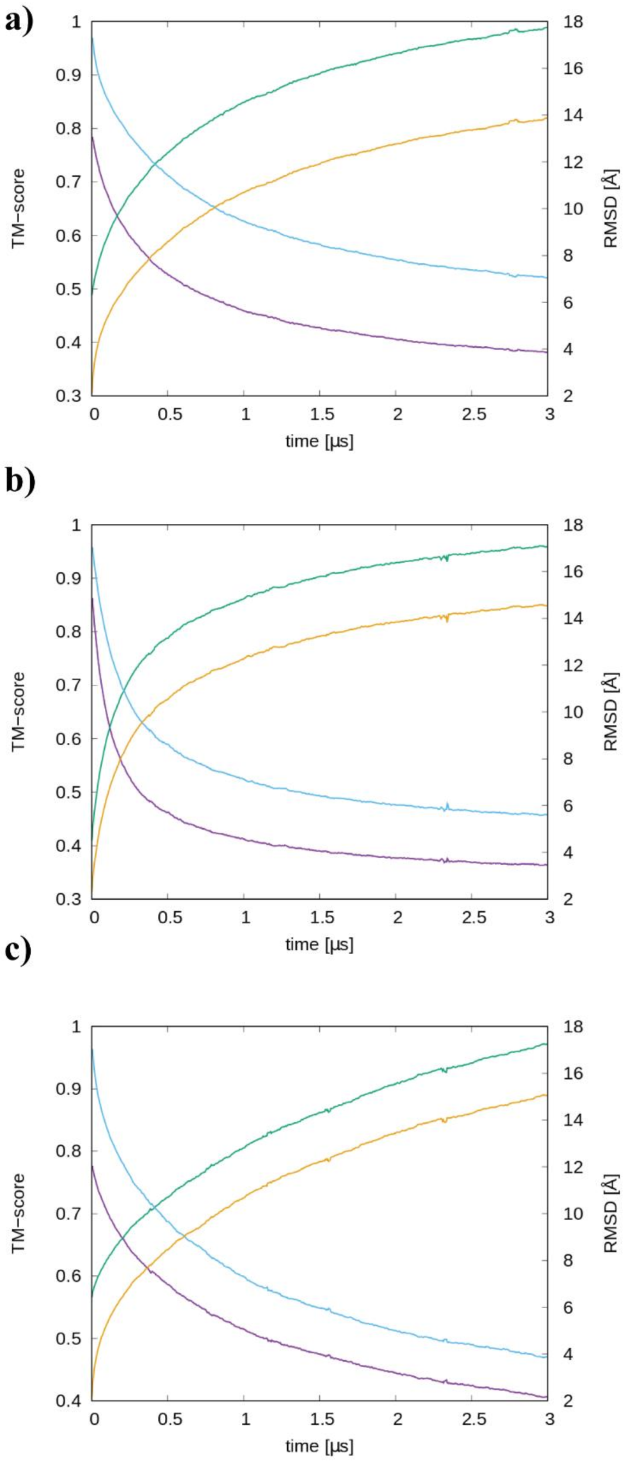

Figure S5. UNitedRESidue (UNRES) coarse-grain simulations of the stab-1 HCC oligomers at 277K.

The root-mean-square deviation (RMSD) and TM-scores for **a)** dodecamer, **b)** decamer Model 1, and **c)** decamer Model 2 at 277K over all trajectories. The blue and violet lines represent the TM-score plots for the UNRES minimized and original (reference) model, respectively. The yellow and green lines represent the RMSD plots for the UNRES minimized and original (reference) model, respectively.

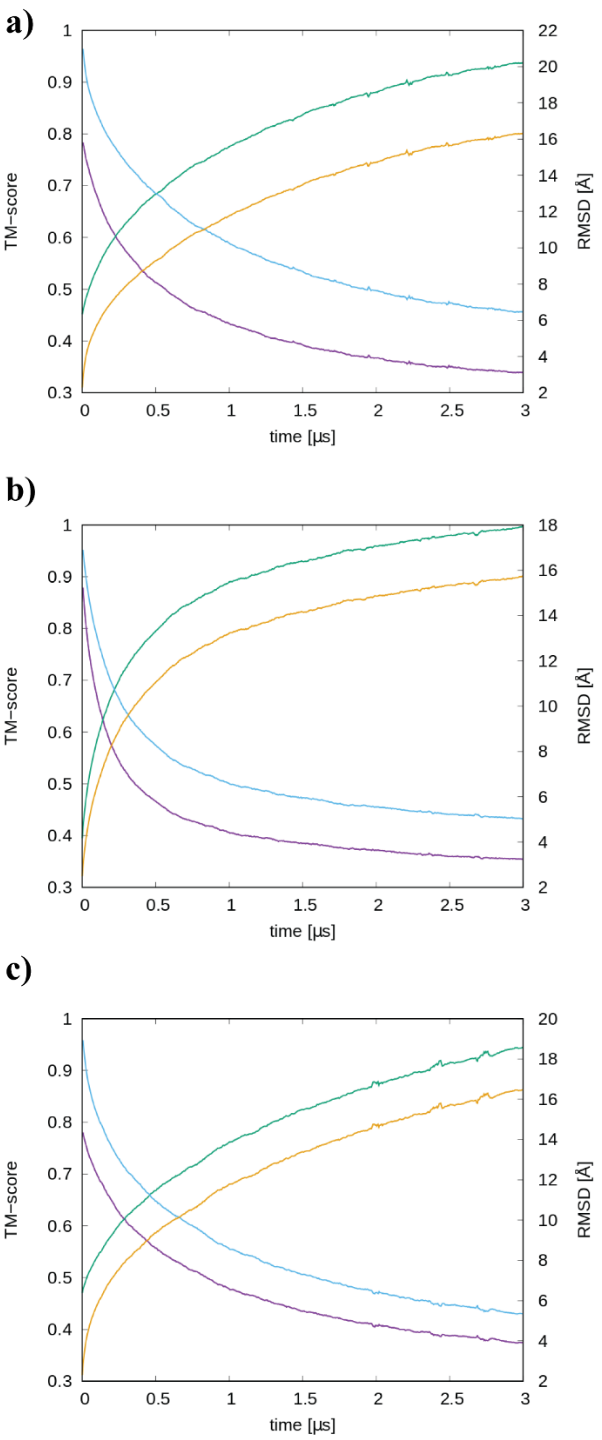

Figure S6. UNitedRESidue (UNRES) coarse-grain simulations of the stab-1 HCC oligomers at 300K.

The root-mean-square deviation (RMSD) and TM-scores for **a)** dodecamer, **b)** decamer Model 1, and **c)** decamer Model 2 at 300K over all trajectories. The blue and violet lines represent the TM-score plots for the UNRES minimized and original (reference) model, respectively. The yellow and green lines represent the RMSD plots for the UNRES minimized and original (reference) model, respectively.

a)

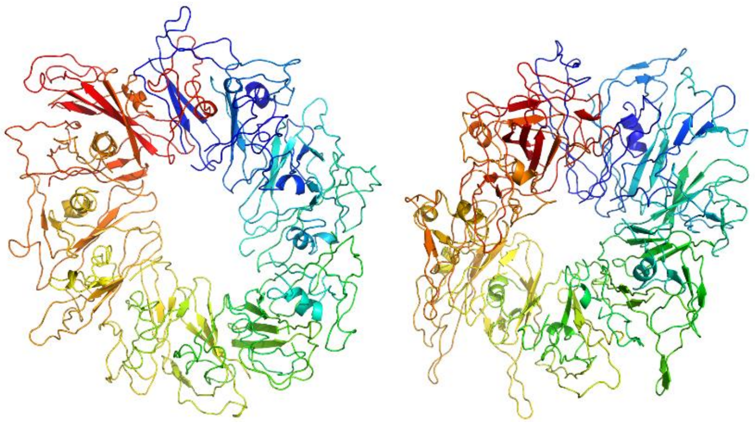

b)

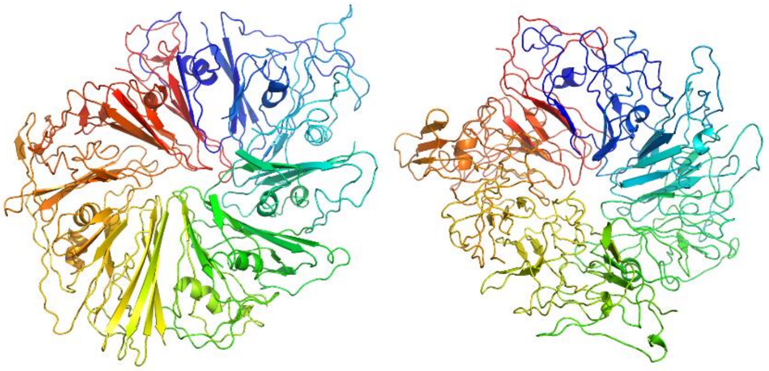

c)

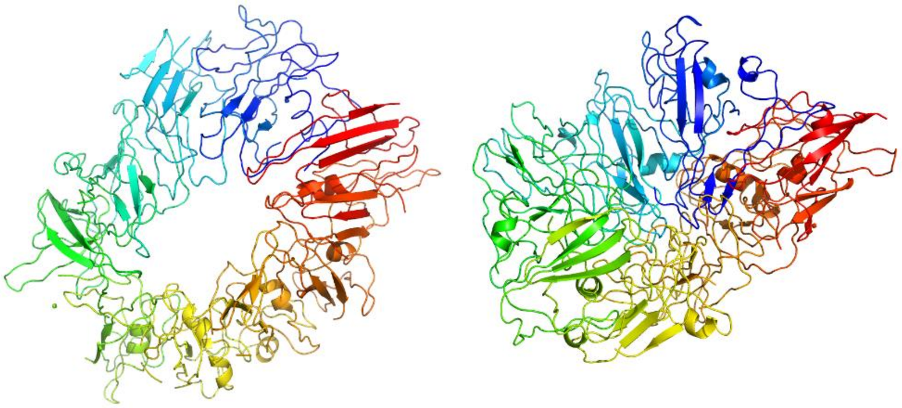

Figure S7. UNitedRESidue (UNRES) coarse-grain simulation models of the stab-1 HCC oligomers at 277K

Representative models after long-term UNRES coarse-grain simulations **a)** dodecamer, **b)** decamer Model 1 and **c)** decamer Model 2 at 277K.

a)

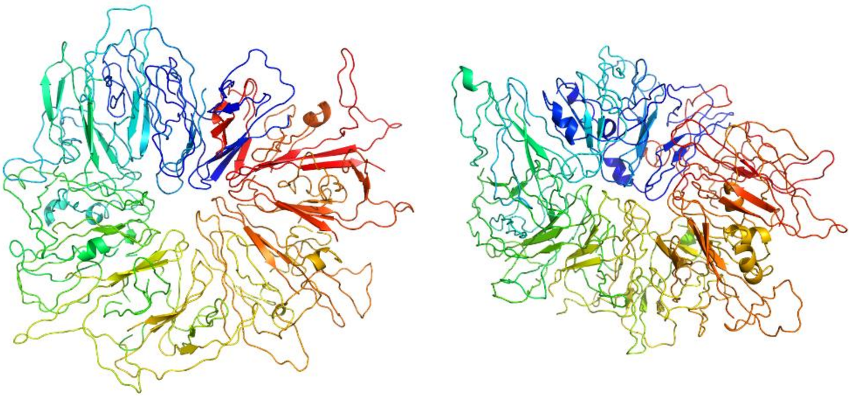

b)

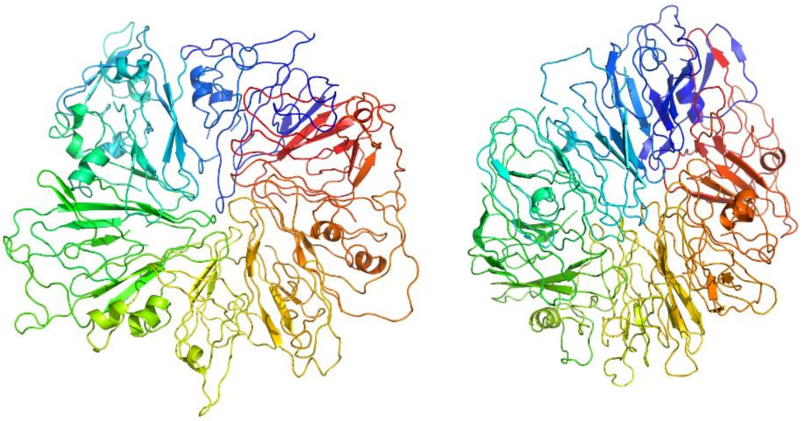

c)

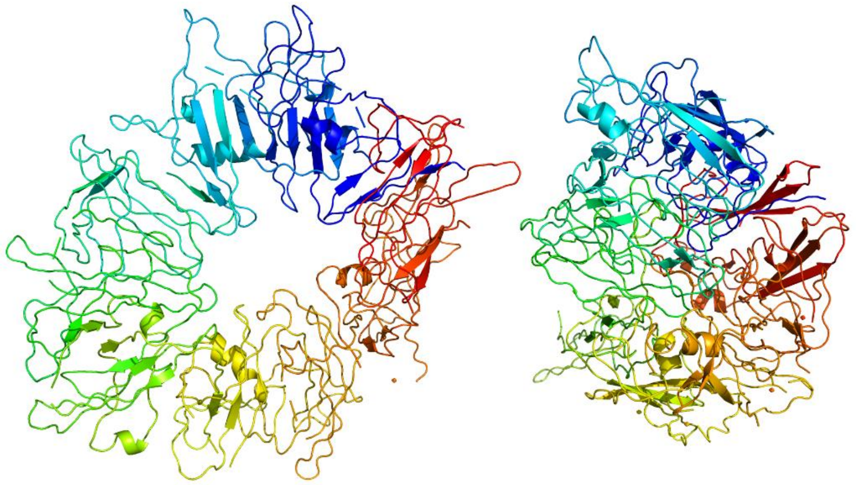

Figure S8. UNitedRESidue (UNRES) coarse-grain simulation models of the stab-1 HCC oligomers at 300K

Representative models after long-term UNRES coarse-grain simulations **a)** dodecamer, **b)** decamer Model 1 and **c)** decamer Model 2 at 300K.

a)

b)

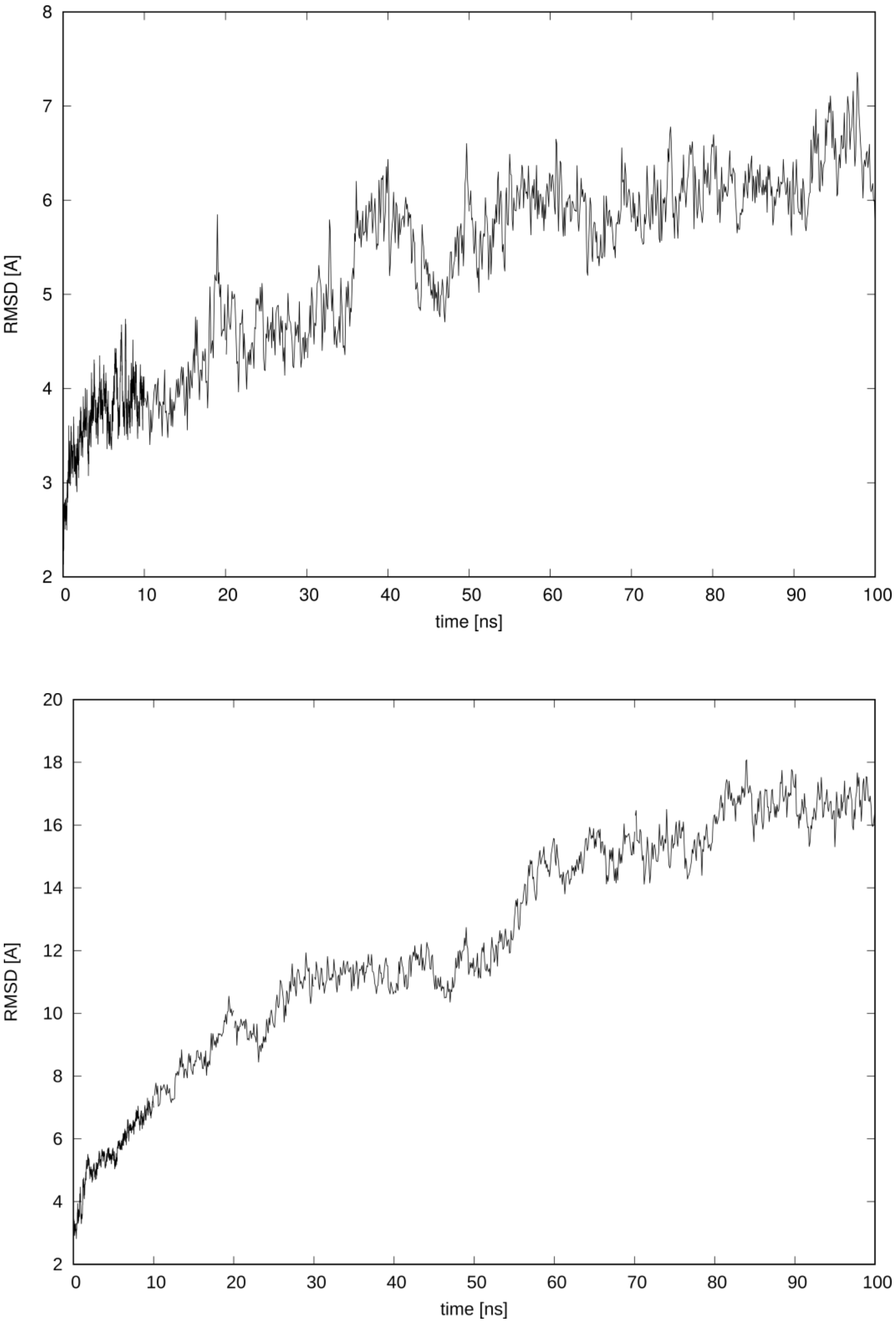

Figure S9 RMSD as a function of time in UNitedRESidue (UNRES) coarse-grain simulations for a) Model 1 decamer and b) dodecamer.

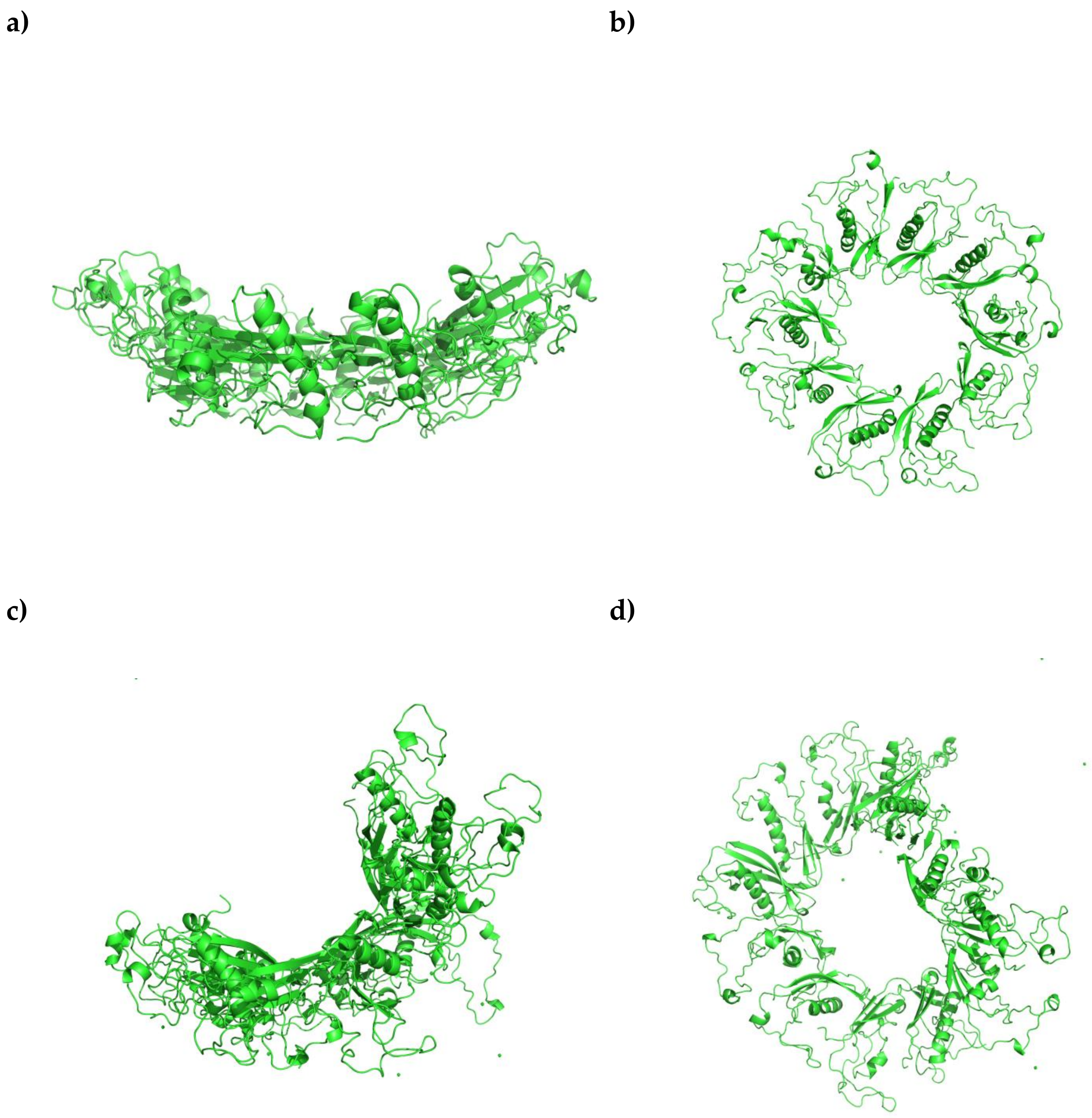

Figure S10 Structure after 100ns coarse-grain simulation of Model 1 of decamer.

a) side view and **b)** top view compared with structure obtained after 100ns simulation of dodecamer **c)** side view and **d)** top view.
